# Building the capacity of Sierra Leoneans in supply chain on the National Pharmaceutical Procurement Unit (NPPU) project (a case study)

**DOI:** 10.1186/2052-3211-7-S1-O25

**Published:** 2014-12-17

**Authors:** Maurice Juma, Jack Lansana, Francis Dawoh

**Affiliations:** 1 Crown Agents, Freetown, Sierra Leone; 2 National Pharmaceutical Procurement Unit (NPPU), Freetown, Sierra Leone

## Background

A need to strengthen the supply chain and capacity of local supply chain professionals in Sierra Leone was identified following a supply chain assessment in 2010. In 2012, Crown Agents was contracted to undertake a project to set up and manage the National Pharmaceutical Procurement Unit project and build local capacity over a 3 year period. The project team consists of international supply chain professionals and their Sierra Leonean counterparts to whom they are tasked with building capacity.

## Method

The project team implemented a detailed capacity development plan, designed specifically to meet the individual development needs of the local Sierra Leonean counterpart executives. Each development plan was tailored to ensure that the counterparts’ capacities were built through mentoring, on the job training, attendance on accredited external professional training courses, regular monitoring and evaluating. Additionally, capacity development to strengthen the existing non-executive workforce in other department was also delivered.

## Results

The counterparts received specific “on the job” training and learning which they were able to confidently apply to everyday situations in order to make significant improvements to the medical supply chain. Additionally counterparts attended external supply chain specific accredited courses in procurement and supply chain management. The mentoring was useful as it taught the counterparts how to meet challenging workloads and effective liaise with people at all levels from teams that they may manage to development partners and officials in various government ministries.

## Discussion

During the project’s implementation the counterpart management team received effective capacity development to allow them to undertake their specific supply chain roles with confidence and provide effective support to their management team. The mentoring programme meant that learning and development was always available and the counterparts were able to gain firsthand experience of planning approaches, meeting deadlines and effective management in supply chain on a daily basis. Additionally the counterparts gained exposure to other areas of supply chain management including stakeholder relations.

## Lessons learned

It is important to undertake an initial comprehensive assessment of the development requirements of the counterparts in order to plan the development plan to be implemented. It is important to review this plan regularly with the counterpart to see if any changes may need to be made to address any new development areas.

